# Supplementary material for: Mixbiotic society measures: Assessment of community well-going as living system
Source: PLoS One. 2024 Aug 7;19(8):e0307401. doi: 10.1371/journal.pone.0307401 (PMC11305589; doi:10.1371/journal.pone.0307401)
Supplement: Attachment — Submitted filename: Rebuttal_letter_1202.docx [file pone.0307401.s001.docx]

December 2, 2023

The Editorial Board

*PLOS ONE*

Dear Editor and Reviewers:

Thank you for your suggestions and comments on our manuscript titled “**Mixbiotic society measures: Assessment of community well-going as living system**.” We also appreciate the time and effort you and each of the reviewers have dedicated to providing insightful feedback on ways to strengthen our paper. We have incorporated changes that reflect the detailed suggestions you have so graciously provided. We hope that our edits and the responses provided below satisfactorily address all the issues and concerns you and the reviewers have noted.

To facilitate your review of our revisions, the following is a point-by-point response to the questions and comments delivered in your letter [PONE-D-23-27480].

**Response to Editor:**

1. The attempt to introduce a mixbiotic social theory presents fundamental flaws that require a complete rethinking and rewriting of the paper. Some aspects of the model (in particular, the communication network) and of the algorithm are not well formalized, are obscure or inconsistent.

**Response:** Thank you for helpful suggestion. The network and communication models in the Methods section were for planning our measures, and the establishment of the models themselves was not the claim of this paper. Therefore, we have omitted the items on these in the Methods section and the items on network formation and communication simulation in the Results section.

2. The underlying idea of the paper very interesting and I believe that your mixbiotic social theory has potential for real-world applications.

**Response:** Following your advice, we made the changes to the sentense structure as the above and revamped the manuscript with real-world applications as the main focus. Furthermore, as suggested by the reviewer #1, we have added a new Literature Review and Our Approach section and moved part of the previous Introduction section to this section.

**Response to Reviewer #1:**

1. The authors rewrite the abstract and the introduction, briefly introducing the concepts.

**Response:** Thank you for this suggestion. We have added the description of “social isolation” (p. 3, lines 51–53) and the description of “social fragmentation” (p. 3, lines 56-59).

2. Where “community impoverishment” come from (who used firstly the term “atomism”?) needs to be clarified.

**Response:** Thank you for this suggestion. We have added the explanation (p. 4, lines 82–84).

3. It is suggested that a section be devoted to a detailed review of the literature.

**Response:** Thank you for this suggestion. As written in our response to the editor above, we have added a new Literature Review and Our Approach section (p. 5, lines 133).

4. Show the meaning of “communication network.”

**Response:** Thank you for this suggestion. We have added the explanation (p. 5, lines 105–107).

5. Comment on “among people in primary and high schools or in a Malawi village only the interactions are recorded, no communication is necessarily exchanged.”

**Response:** Thank you for this suggestion. We have added the explanation (p. 15, lines 311–314).

6. Say explicitly somewhere in the manuscript that Q is a vector and q_i are its component.

**Response:** Thank you for this suggestion. We have added the description (p. 11, lines 236–239).

7. The lack of necessary details: the step from Eq (4) to Eq (5), the distribution of n_0, the functional form of f_0, the rounding function, and t_max.

**Response:** Thank you for checking it out in detail. As written in our response to the editor above, we have omitted the network and communication models in the Methods section.

8. There is no sence in writing the paragraph about strange attractor without without any kind of analysis of the chaotic regime of your model.

**Response:** Thank you for this suggestion. We have omitted this paragraph.

9. The application to real data is quite promising.

**Response:** Thank you for this advice. As written in our response to the editor above, we have revised the manuscript with a focus on the application to real data.

**Response to Reviewer #2:**

1. The communication model lacks a comprehensive description and formalisation. Answer the questions on why you have selected this specific communication model and why you expect that this simple one would give rise to the expected scenarios as in CA and PRD.

**Response:** Thank you for this suggestion. As written in our response to the editor above, the communication model were for planning our measures, and the establishment of the model corresponding to CA and PRD was not the claim of this paper. Threfore, we have omitted the item on communication model.

2. The authors have highlighted the “edge of chaos” as an intriguing phase characterised by a balance between order and disorder. This idea can be extrapolated to the social and anthropological interpretation of the livingness of an autopoietic society, between freedom and solidarity.

**Response:** Thank you for this advice. We have reflected this comment (p. 9, lines 198–200).

3. The section on the communication model suffers from inadequate writing: Q —> Q in Eq (5), f0, fe, fr, and Eq (8).

**Response:** Thank you for checking it out in detail. As written in the above, we have omitted the communication model in the Methods section.

4. Mention the recent works on temporal networks or high-order network which would surely be more interesting to address processes among community.

**Response:** Thank you for this suggestion. We have added the recent works (p. 7, lines 149–152, lines 157–165).

5. WS and BA models have severe limitations, specifically in recreating realistic community structure.

**Response:** Thank you for this suggestion. As written in the above, we have omitted the communication model.

6. Provide basis that the proposed approach is promising and explain why the concepts valid in the simple CA and PRD models can be translated to your specific model.

**Response:** Thank you for this suggestion. Rather than trying to translate the CA and PRD models into our communication model, we have extrapolated that idea into a social interpretation as inspiration (p. 9, lines 198–200).

7. The last 5 years have seen the emergence of several method for temporal networks as they are fundamental to study epidemics and information spread.

**Response:** Thank you for this suggestion. As written in the above, we have added the recent works (p. 7, lines 149–152, lines 157–165).

8. AC components are unknown.

**Response:** Thank you for this suggestion. We have rewritten it (p. 9, lines 193–194).

9. Table 1 is not easy to unroll.

**Response:** Thank you for this suggestion. We have agreed and deleted this Table.

10. Matters pointed out concerning the following: network formation, communication generation, calculation results for communication models, and communication trajectories for communication models.

**Response:** Thank you for your detailed remarks. As written in the above, we have omitted the communication model.

11. This section of real world dataset is probably the most clear, however some of the networks are large compared to the ones were the communication model has been developed.

**Response:** Thank you for this suggestion. Since we omitted the communication model, we do not consider this point to be applicable to the real world data set.

12. The authors didn’t analyse an online social interaction network.

**Response:** Thank you for this suggestion. Actually, we were analyzing an online social interaction network. We have added a specific name to make this clearer (p. 16, line 325).

Thank you, once again, for giving us the opportunity to strengthen our manuscript with your valuable comments and queries. We have worked hard to incorporate your feedback and hope that these revisions persuade you to accept our resubmission.

Sincerely,

Takeshi Kato

Hitachi Kyoto University Laboratory, Open Innovation Institute, Kyoto University

Yoshidahonmachi, Sakyo-ku, Kyoto-shi, Kyoto 606-8501, Japan

+81-75-753-9716

kato.takeshi.3u@kyoto-u.ac.jp
