# Supplementary material for: Mixbiotic society measures: Assessment of community well-going as living system
Source: PLoS One. 2024 Aug 7;19(8):e0307401. doi: 10.1371/journal.pone.0307401 (PMC11305589; doi:10.1371/journal.pone.0307401)
Supplement: Attachment — Submitted filename: Response_to_Reviewers.docx [file pone.0307401.s002.docx]

June 8, 2024

The Editorial Board

*PLOS ONE*

Dear Editor and Reviewers:

Thank you for your valuable suggestions and comments on our manuscript titled “**Mixbiotic Society Measures: Assessment of Community Well-being as a Living System**.” We appreciate the time and effort you and each reviewer have dedicated to providing insightful feedback on ways to strengthen our paper. We have made changes that reflect your detailed suggestions. We hope our edits and responses below satisfactorily address all the issues and concerns you and the reviewers have noted.

To facilitate your review of our revisions, the following is a point-by-point response to the questions and comments delivered in your letter [PONE-D-23-39776].

**Response to Editor:**

1. Clear indication/presentation of study methodology and results both in the abstract and main text.

**Response:** Thank you for your helpful suggestion. We have improved this aspect in response to both your feedback and that of reviewers #1–#3.

2. Logical consistency.

**Response:** We believe this has also been improved.

3. Minor grammatical corrections such as usage of ‘Conversely’ in the fourth sentence of the Introduction instead of ‘On the other hand,’ since the two significant issues in contemporary society.

**Response:** Thank you. We have retained this word (p. 3, line 57).

4. Lesser number of references, to include only relevant ones.

**Response:** The number of references has been reduced from 75 to 65, retaining only the most relevant ones.

5. Presenting study methodology under the ‘Methods’ section, as against describing methodology in the opening sentences of the Result Section.

**Response:** The methodology has been moved from the opening of the Results section to the Methods section (pp. 12–13, lines 315–336).

6. Appropriate caption of the conclusion section to reflect the content of the section, as also seen in the last sentence of the Introduction section.

**Response:** We have added the appropriate caption (p. 19, lines 513–514).

7. Complete and accessible reference sources as contained in the URL. That is, some URL were not accessible such as the 8th list of reference.

**Response:** We have verified and confirmed all URLs.

8. Authors are therefore advised to simplify the texts for common readers to benefit, clearly explaining its relevance, implications and recommendations.

**Response:** We have revised the text following your and the peer reviewers’ advice; we believe the paper is more readable now.

**Response to Reviewer #1:**

1. Throughout the text, different objectives are mentioned (line 30, line 115, line 200, line 211) and none of them clearly describes the objective of the article, which was: to develop, present and apply innovative measures of the mixbiotic society to evaluate dynamic communication patterns , complementing the traditional static analysis of social networks. Furthermore, the objective must be included in the introduction.

**Response:** Thank you for this suggestion. We have revised the study objective and ensured that it is consistently presented in the text (p. 2, lines 28–33, p. 4, lines 97–103).

2. The justification must also be included in the introduction. It is possible to use the text from lines 213 to 233.

**Response:** The justification has been moved from Literature review section to Introduction section (pp. 4–5, lines 108–119).

3. On the text (line 306): “To validate the mixbiotic society measures, we performed calculations on seven temporal network datasets with potentially different communication pattern features.” The data in this study is enough to validate it?

**Response:** We have added the reasons why we chose these datasets and why they are sufficient to validate different communication patterns (p. 13, lines 337–346).

4. The text from lines 308-327 must appear in methods and also in the abstract.

**Response:** As mentioned in our response to the editor, these parts have been moved to the Methods section and added to the Abstract (pp. 12–13, lines 315–336, p. 2, lines 41–42).

5. The sample used in the study should be mentioned on the abstract.

**Response:** We have mentioned the sample (p. 2, lines 41–42).

**Response to Reviewer #2:**

1a. From lines 87 to 108, the authors demonstrate different scholars’ perspectives about the social system. These theoretical backgrounds help readers understand the relationship between individuals and communities. However, these parts should be included in the literature review.

**Response:** Thank you for this suggestion. These parts has been moved from Introduction section to Literature review section (pp. 5–6, lines 133–153).

1b. The The authors didn’t mention nihilism until line 293, but the concept is in the abstract, so it should be introduced earlier in the literature review section to avoid confusion.

**Response:** We have mentioned it in Introduction before Literature review section (p. 4, line 106).

1c. The authors discuss their datasets starting from line 308, and this should be included in the method section.

**Response:** As mentioned in our response to the editor and reviewer #1 above, we have included these parts in Methods section (pp. 12–13, lines 315–336).

2. The authors use different settings, e.g., high school, primary school, workplace, etc., in their dataset without giving justifications. How do these diverse settings contribute to the overall research objectives? How do the mixbiotic society measures gauge the activities in different settings?

**Response:** As mentioned in our response to reviewer #1 above, we have added the reasons why we chose these datasets and why they are sufficient to validate the mixbiotic society measures (p. 13, lines 337–346).

3. It would be helpful if the authors could explain more about the application of this dynamic network analysis versus the traditional static analysis in the conclusion section. For example, they wrote: “the mixism measure can be utilized as a measure of balanced well-going between similarity and dissimilarity, proximate mixing and diverse mingling toward a desirable mixbiotic society. Combining the present measures with the structural analysis of social network and multiscale entropy analysis of temporal network will provide more insights”. It would be worth expanding the potential implications in real-world settings.

**Response:** We have added how mixbiotic society measures in combination with static social network analysis or multiscale entropy analysis can have real-world significance (p. 20, lines 532–538).

4. The authors are not very clear about how introducing the methods of CA and PRD can better measure the dynamic perspective of network analysis in the literature section. What is the connection between these methods? I wish I could see more elaborated explanations in the paragraph starting with line 198.

**Response:** We have revised the explanation of what MSE, CA, and PRD can suggest in measuring the dynamic perspective of network (p. 8, lines 216–220).

**Response to Reviewer #3:**

1. The clarity of the idea and the information is not precisely focused, and how is it possible to connect isolation, fragmentation, and the mixed neighborhood? I think the writer should expand on that.

**Response:** Thank you for this suggestion. As mentioned in our response to reviewer #2 above, we have added the explanation of how mixbiotic society measures, along with social network analysis or multiscale entropy analysis, are related to isolation and fragmentation (p. 20, lines 532–538).

Thank you, once again, for providing us the opportunity to strengthen our manuscript with your valuable comments and queries. We have worked hard to incorporate your feedback and hope that these revisions persuade you to accept the revised manuscript for publication.

Sincerely,

Takeshi Kato

Hitachi Kyoto University Laboratory, Kyoto University

Yoshidahonmachi, Sakyo-ku, Kyoto-shi, Kyoto 606-8501, Japan

+81-75-753-9716

kato.takeshi.3u@kyoto-u.ac.jp
